# TITAN: Combining a bidirectional forwarding graph and GCN to detect saturation attack targeted at SDN

**DOI:** 10.1371/journal.pone.0299846

**Published:** 2024-04-26

**Authors:** Longyan Ran, Yunhe Cui, Jianpeng Zhao, Hongzhen Yang

**Affiliations:** 1 Guizhou Xiangming Technology Co., Ltd, Guiyang, Guizhou, China; 2 State Key Laboratory of Big Data, College of Computer Science and Technology, Guizhou University, Guiyang, Guizhou, China; 3 State Grid Zhejiang Electric Power Co., Ltd, Information and Telecommunication Branch, Hangzhou, Zhejiang, China; Universiti Malaysia Sabah, MALAYSIA

## Abstract

The decoupling of control and forwarding layers brings Software-Defined Networking (SDN) the network programmability and global control capability, but it also poses SDN security risks. The adversaries can use the forwarding and control decoupling character of SDN to forge legitimate traffic, launching saturation attacks targeted at SDN switches. These attacks can cause the overflow of switch flow tables, thus making the switch cannot forward benign network traffic. How to effectively detect saturation attack is a research hotspot. There are only a few graph-based saturation attack detection methods. Meanwhile, the current graph generation methods may take useless or misleading information to the attack detection, thus decreasing the attack detection accuracy. To solve the above problems, this paper proposes TITAN, a bidirec**T**ional forward**I**ng graph-based satura**T**ion **A**ttack detectio**N** method. TITAN defines flow forwarding rules and topology information, and designs flow statistical features. Based on these definitions, TITAN generates nodes of the bi-forwarding graph based on the flow statistics features and edges of the bi-forwarding graph based on the network traffic routing paths. In this way, each traffic flow in the network is transformed into a bi-directional forwarding graph. Then TITAN feeds the above bidirectional forwarding graph into a Graph Convolutional Network (GCN) to detect whether the flow is a saturation attack flow. The experimental results show that TITAN can effectively detect saturation attacks in SDNs with a detection accuracy of more than 97%.

## 1 Introduction

Software-Defined Networking (SDN) is an emerging network architecture that brings great changes and enhancements to traditional networks. SDN is consisted of the application layer, northbound interface, control layer, southbound interface, and forwarding layer. The application layer consists of various network services and applications. The control layer is responsible for controlling network traffic while the forwarding layer only needs to forward network traffic under the instructions made by the control layer. At the same time, applications in the application layer can utilize various network services provided by the control layer through the northbound interface. Multiple controllers can work together through the east-west interface. SDN has network programmability and good scalability in both vertical and horizontal directions. Therefore, SDN is widely used in cloud data center networks [1], Internet of Things (IoT) [2], and blockchain [3].

The separation of forwarding and control function enables SDN to be widely used in some areas, but it also poses security risks to SDN. Specially, adversaries can exploit the unique forwarding and control separation mechanism to launch saturation attacks. Due to the high cost and power consumption of Ternary content-addressable memory (TCAM), SDN switches can only install a limited number of flow entries [4]. The adversaries can utilize the limited TCAM of SDN switches to launch saturation attacks targeted at overwhelming the SDN switches [5]. More specifically, they can send elaborated attack packets to the target switch, causing the switch to install many flow entries. Eventually, the above flow entries exhaust the TCAM memory and cause the SDN switch fail to install new forwarding rules, thus disrupting the transmission of benign traffic. For instance, Zhang et al. [6] proposed an enhanced saturation attack Table-miss Striking Attack, in which the adversary first uses various low-rate probe packets to probe the detailed information of flow entries, especially for the match field and survival time. Based on these information, it forges some attack packets to inveigle the switch send *packet-in* messages to the controller. Then the controller will send a lot of *flow-mod* messages to install flow entries in the switch. At last, the switch will be occupied by the malicious flow entries.

How to detect saturation attacks is a serious challenge in SDN. Some researchers use machine learning-based approaches to detect network attack [7]. Due to its high detection accuracy, a few studies have applied Graph Neural Networks (GNN) to DDoS attack detection in SDNs. For instance, by mapping the SDN network as a graph, the method proposed in [8] first identifies the switches under DDoS attack, and then precisely locates the entrance switch of attack flow. The literature [9] also maps SDN smart grid as graphs, but it firstly detects whether the network is anomalous and then identifies the phasor measurement unit that suffer DDoS attacks. Meanwhile, some researchers have applied Graph Attention Networks (GAT) to anomaly detection in sensor networks to identify anomalous sensor nodes [10]. The detection objects in the literature [9, 10] are network nodes and it do not discriminate the traffic. The literature [11] transforms the session instances into K-Nearest Neighbors (KNN) graphs and uses the GCN model to detect each KNN graph. The KNN graph in [11] is generated by calculating the Euclidean distance between features of different instances. Then the *k* nearest nodes is selected to add edges for the central node. When using KNN graph to detect SDN saturation attack, it needs to convert each network flow into a KNN graph. GCN-TC [12] uses traffic trace graph to represent traffic flows. In traffic trace graph, nodes are traffic flows, and edges denote that nodes have common IP hosts. When using GCN-TC to detect SDN saturation attack, edges will be added among nodes that have common IP hosts, regardless of the type of nodes. Hence, it may make the GNN model aggregate attack node information using incorrect neighbor nodes, causing the decrease of detection performance.

In conclusion, although GNN can achieve high detection accuracy, there are very few graph-based saturation attack detection methods in SDN. Meanwhile, the existing graph-based saturation attack detection methods are facing with the following issues.

- **Issue 1: the edges generated by existing graph generation methods maybe incorrectly connected**. When using KNN graph, the features are normalized as nodes, and it adds edges based on the Euclidean distances among different nodes. Such an edge does not carry useful information and only indicates that a few types of features are similar in value. In addition, the traffic trace graph adds edges depending on whether the nodes have a common IP host. Hence, traffic trace graph may generate edges connected the normal flow nodes and attack flow nodes. These edges may take incorrect information to GNN model.
- **Issue 2: incorrectly connected edges may have negative effect on aggregating nodes information of GNN**. It is known that the statistical characteristics of normal flow nodes and attack flow nodes are quite different. When GNN model aggregates the nodes information using traffic trace graph, the incorrectly connected edges among normal and attack nodes will make GNN aggregate the attack nodes information based on the normal nodes information. Hence, GNN will weaken the difference between these two kinds of nodes instead of enhancing it. In conclusion, the incorrectly connected edges will decrease the detection accuracy.

To solve the above problem, this work focuses on how to detect saturation attack flows against OpenFlow switches. Hence, we propose TITAN, a bidirec**T**ional forward**I**ng Graph and GCN-based sa**T**uration **A**ttack detectio**N** method. TITAN designs a bidirectional forwarding graph generation algorithm, by defining the notion of topology information and flow forwarding rules and designing flow statistical features. The bidirectional forwarding graph generation algorithm transforms each flow in the SDN network into a bidirectional forwarding graph. Meanwhile, a GCN model including three graph convolutional layers, one fully connected layer, and one classification module is designed to identify whether the bidirectional forwarding graph belongs to saturation attack.

Main contributions of this work are listed as follows.

- This work proposes TITAN, a bidirectional forwarding graph and GCN-based saturation attack detection method. TITAN designs a bidirectional forwarding graph generation algorithm to convert each flow into a bidirectional forwarding graph, and then it uses GCN to implement the detection of saturation attack flows in SDNs.
- As far as we know, TITAN is the first work that converts all information about a network flow into a single graph to detect saturation attack flow. When generating the bidirectional forwarding graph, TITAN constructs node information based on the topology and flow statistics features. Meanwhile, the edge information is generated based on the topology information and flow forwarding rules.
- TITAN designs a GCN model consisted of three graph convolutional layers, one fully connected layer, and one classification module to detect the bidirectional forwarding graph.
- The evaluation results show that TITAN can effectively detect saturation attack flows in SDNs.

The remainder of this paper is organized as follows. Section 2 investigates the state-of-the-art works on saturation attack detection and graph-based attack detection. The background is introduced in Section 3. Section 4 presents the detailed architecture of TITAN. The evaluation is shown in Section 5. Section 6 summarizes the conclusions of this work.

## 2 Related works

Section 2 gives the description of related works. It first summarises the graph-based attack detection methods in subsection 2.1, and then describes the current SDN saturation attack detection methods in subsection 2.2. Meanwhile, at the end of subsection 2.2, we analyze the differences among the proposed method and other graph-based network attack methods.

### 2.1 Graph-based attack detection method

Cao et al. used in-band network telemetry to obtain the device state of the SDN data layer and map the data layer into a graph [8]. To identify whether a switch is passed by DDoS flows, this method employs a ST-GCN (Spatio-Temporal Convolutional Graph Convolutional Network) to detect DDoS attack. Finally, a defense strategy is proposed based on the attack detection result. The author in [9] proposed a DDoS detection method in SDN smart grid environment called GLASS. GLASS maps the SDN smart grid into an undirected graph by calculating the transmission delay between adjacent phasor measurement units (PMU), the average transmission delay of a central PMU and its all neighboring PMUs as the edge weights and the weight of the central PMU node, respectively. GLASS is divided into two phases, first determining whether the SDN smart grid is under DDoS attack using a GCN and then using a spectral clustering algorithm to determine the PMU under attack. Some researchers have used the graph attention mechanism for anomaly detection in sensor networks [10]. This method uses nodes to represent sensors, calculates the cosine similarity between nodes using their feature vectors and adds edges using the TOPK algorithm to generate the graph. Subsequently, the anomalous sensor nodes in the graph are identified with GAT. Liu et al. [11] proposed a graph generation approach to create knn graphs for each flow instance. This approach uses nodes to represent features, calculates the Euclidean distance between features, and adds undirected edges between nodes based on the k-nearest neighbor algorithm. Finally the attack detection is done using GCN for graph classification. GCN-TC, proposed in [12], uses traffic trace graph to classify network flows. In traffic trace graph, nodes represent traffic flow, edges denote that nodes have common IP hosts. After generating the traffic trace graph, GCN-TC uses GCN model to classify a traffic trace graph belongs to different kinds.

These works [8, 9] are multi-stage detection methods with coarse grained followed by fine grained. The multi-stage detection method has a problem of long detection time. In particular, the GLASS can only identify the attacked switch. Additionally, implementing mitigation strategies based on the attacked switches may have an impact on normal traffic. The work in [10] suffers the same issue as the GLASS. The approach in [11] takes a more fine-grained approach and creates graphs for each session. The traffic trace graph in [12] has the problem that it may connect normal and attack nodes, resulting in low detection accuracy. However, graph data occupies more memory than text, which may slow down model training and detection. In conclusion, GNN-based methods can be effectively used in attack detection. However, many current approaches have limitations, like some studies have been confined to the graph node classification problem for the application of GNN.

Main differences between TITAN and the existing graph-based network attack detection methods are shown in Table 1. The main limitation of the existing graph-based network attack detection methods is that the graphs generated by these works may lead to incorrect connections between different nodes, which makes GNN model aggregate node information based on incorrect neighbor nodes. Therefore, the detection accuracy of these methods maybe decreased. To overcome the above issue, this work proposes TITAN. TITAN designs a graph information preprocessing method, a bidirectional forwarding graph construction method and a Bifor-Graph-based saturation attack detection method to detect saturation attacks in SDN.

**Table 1:** Main differences between TITAN and graph-based network attack detection methods.

Reference	Application scenarios	Technique	Comments
[8]	DDoS detection.	Topology graph; ST-GCN.	Topology graph maybe unable to precisely represent the traffic flow state.
[9]	SDN-based smart grid DDoS detection.	PMUs graph; GCN.	Device graph may not provide accurate network traffic flow status.
[10]	Network anomaly detection.	TOPK algorithm; GAT.	Sensor graph generated by TOPK algorithm maybe unable to accurately represent the network traffic flow status.
[11]	Intrusion detection.	KNN Graph; GCN.	It may take useless or misleading information to attack detection.
[12]	Traffic flow classification.	Traffic Trace Graph; GCN.	Different types of nodes may influence each other, leading to the decrease of detection accuracy.
TITAN	SDN saturation attack detection.	Bidirectional Forwarding Graph; GCN.	The proposed bidirectional forwarding graph contains comprehensive information of network traffic flows.

### 2.2 Other saturation attack detection method

Li et al. [13] proposed a saturation attack detection method named SA-Detector. It uses the self-similarity of OpenFlow traffic in SDN southbound interfaces to detect saturation attacks. Khamaiseh et al. [14] found that the existing saturation attack detection methods usually acquire network information periodically and only detect saturation attacks caused by TCP-SYN. Hence, these methods cannot detect unknown types of saturation attacks, and they cannot detect saturation attack in time. Therefore, they investigates the variation of OpenFlow traffic triggered by multiple saturation attacks and identify saturation attacks using SVM, NB, and KNN algorithm. Huang et al. [15] analyzed the saturation attack characteristics. When an attack occurs, they found the Packet-In messages rise dramatically and the occupancy of the link bandwidth between each switch and the controller becomes unstable. Thus, the number of Packet-In messages and the entropy value of the control channel occupancy are utilized to detect the attack. The vSwitchGuard [16] is proposed to defend OpenFlow switches against saturation attacks. The authors found that when a saturation attack against switch exist in SDN, a large number of packets cannot be matched with flow entries, causing a large number of Table-miss packets and Packet-In messages. Hence, the saturation attack can be located by calculating the entropy value of the source IP address of Table-miss packets and the proportion of Table-miss packets among all received packets.

In summary, current saturation attack detection techniques heavily rely on the variation of the interaction information between the control and forwarding layer. However, the above methods are lacking the analysis of the variation of flow entries triggered by saturation attacks. Therefore, the above methods can only determine whether the network is under attack or locate the attack target. They cannot identify the saturation attack flow, thus cannot provide enough information for the saturation attack mitigation.

## 3 Background

In this section, we provide the background of this work. Firstly, we introduce the principle of saturation attack against SDN switch in subsection 3.1. Subsequently, subsection 3.2 briefly presents the graph convolutional network.

### 3.1 Saturation attacks targeted at the SDN switch

SDN consists of an application layer, a control layer, and a forwarding layer. SDN switches forward packets based on flow entries. If the switch does not have a flow entry that matches the packet, the SDN switch encapsulates the packet in a Packet-In message and sends it to the controller, and the controller calculates the route based on the packet’s address information. The controller then encapsulates the routing information in a Packet-Out message back to the SDN switch, which installs the flow entry based on the Packet-Out message [17]. This particular control forwarding mechanism raises security threats to the SDN. Saturation attacks exploit this forwarding mechanism to launch attacks on the SDN forwarding layer and disrupt normal network communication. The attacker can attack a legitimate switch through a malicious host, SDN switch TCAM has limited memory and can install limited flow entries. The attacker prompts the switch to install illegal flow entries by sending a large number of invalid packets. When the illegal flow entries occupied the flow table storage of the switch, the forwarding rules matching normal packets cannot be installed, which resulted in the disruption of normal flow in the network. It should be noted that, in this work, we do not consider the saturation attack targeted at the SDN controller [18]. It mainly focuses on detection of saturation attack targeted at the SDN switches.

**Fig 1:**
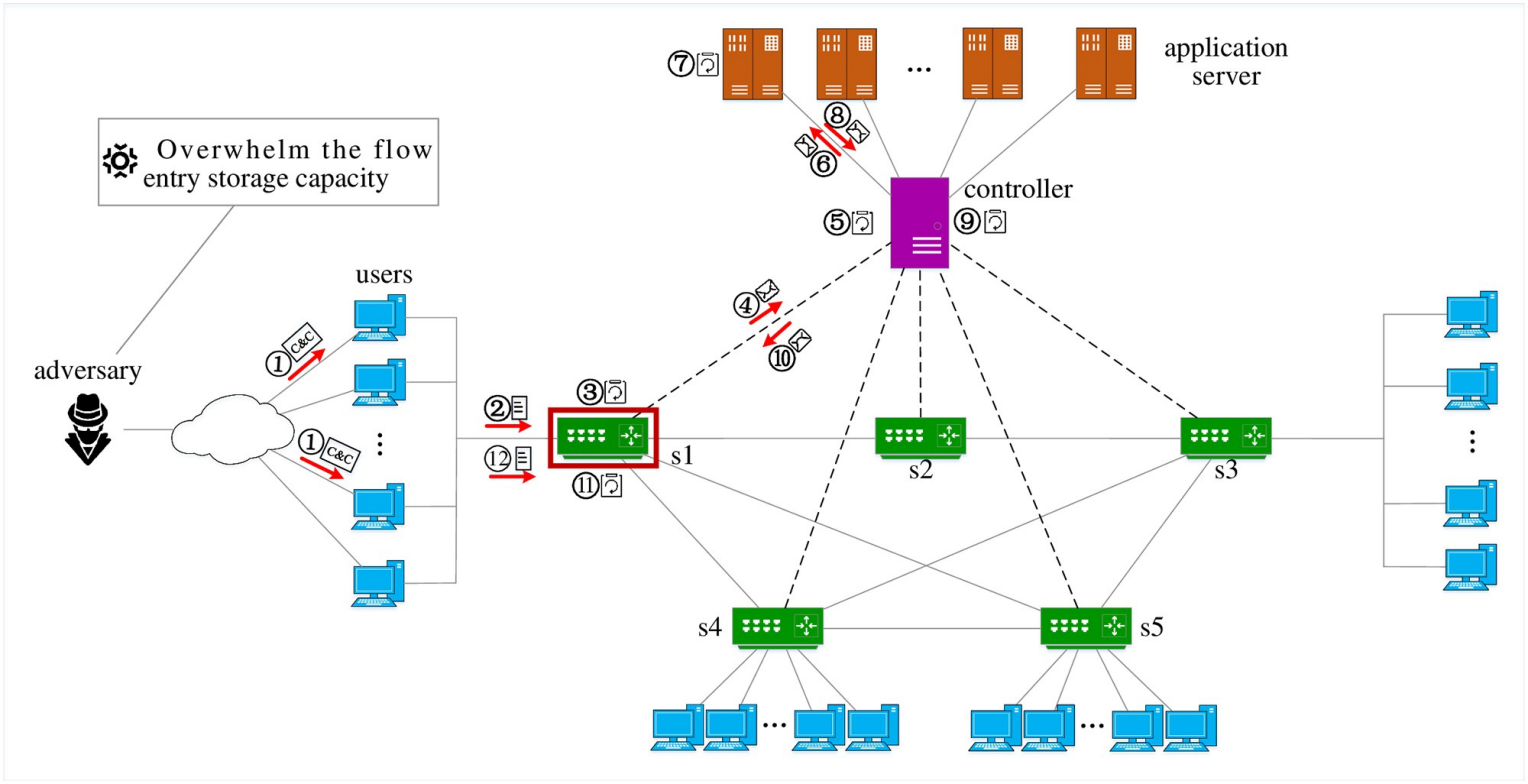
Saturation attack targeted at the SDN switch [17].

In conclusion, the purpose of the saturation attack targeted at the SDN switch is to overwhelm the flow entry storage capacity of the target switch. Fig 1 illustrates the workflow of a saturation attack against the SDN switch, which contains twelve steps listed as follows.

- Step 1 and 2: Controlling zombies. The adversary controls a set of zombies using Control and Command(C&C) messages. Then, the adversary commands these zombies to send saturation attack packets to the targeted switch.
- Step 3: Parsing packets. The targeted switch parses these saturation attack packets, and then it tries to find flow entries that match these packets.
- Step 4: Sending Packet-In messages. The saturation attack packet can not match all flow entries of the target switch. The target switch encapsulates these un-matched packets in the Packet-In messages and sends them to the SDN controller.
- Step 5, 6, 7, and 8: Processing Packet-In messages. The SDN controller parses Packet-In messages generated by un-matched packets and delivers these packets to the related application server. Then, the application server makes the processing decision based on the un-matched packets and returns them to the SDN controller.
- Step 9, 10: Implementing processing decisions. According to the above decisions, the SDN controller encapsulates Packet-Out messages and sends them to the switch to install flow entries.
- Step 11: Installing flow entries. The switch installs flow entries according to the received Packet-Out messages.
- Step 12: Keeping flow entries alive. The adversary makes the zombies continuously send packets that match the newly installed flow entries to keep these flow entries alive on the switch for a long time.

Using the above steps, the adversary can achieve the purpose of overwhelming the flow entry storage capacity of the target switch.

### 3.2 Graph Convolutional Network

Graph neural network (GNN) extends traditional neural networks and enables it to process data represented by graphs [19]. GNN are commonly used for classification and regression tasks. In classification tasks, GNN usually abstract the instances as graphs and extract the features of their nodes or edges as the input of GNN. The final output is the classification result of the node, edge, or graph.

Graph Convolutional Network (GCN) is a generalization of Convolutional Neural Network (CNN) in the graph domain. GCN is a convolutional neural network that can work directly on a graph and exploit its spatial structure information. The GCN aggregates the feature information of each node itself and all its neighbor nodes. Also, the degree of each node in the graph is considered in the aggregation operation to reduce the impact caused by the degree imbalance of different nodes. The final weighted average of the features including the node itself and all its adjacent nodes is obtained, and the relatively optimal graph embedding representation is procured.

In addition, convolution-based graph networks have the advantages of few training parameters and fast training speed [20]. Therefore, GCN is usually applied in medical [21], image classification [22–24], cyber security [25–27], and other fields to perform graph classification tasks.

## 4 TITAN: A bidirectional forwarding graph and GCN-based saturation attack detection method

This section gives the detailed description of TITAN. We firstly present the overview of TITAN in subsection 4.1. Then, in subsections 4.2, 4.3, and 4.4, we present the graph information preprocessing method, bidirectional forwarding graph construction method and the Bifor-Graph-based saturation attack detection method, respectively.

### 4.1 Overview of TITAN

**Fig 2:**
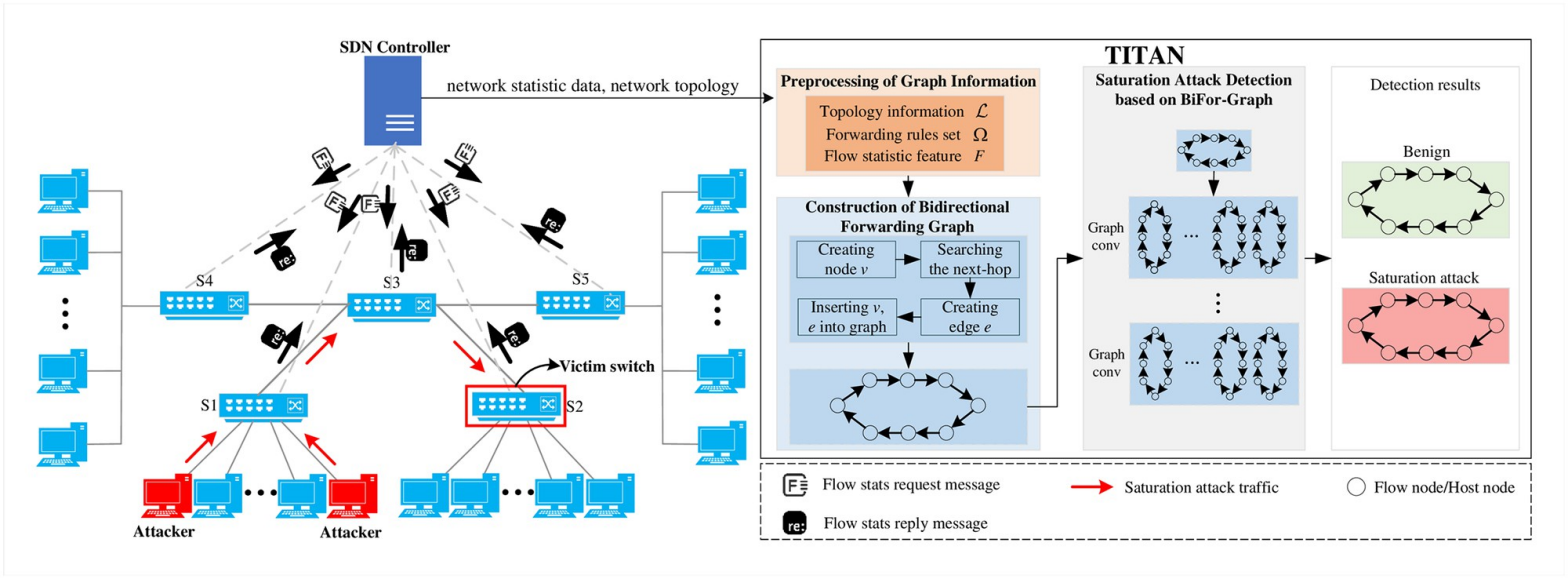
Overview of TITAN.

As shown in Fig 2, this work proposes TITAN, a saturation attack detection method based on bidirectional forwarding graphs and GCN. TITAN consists of three steps: 1) graph information preprocessing, 2) bidirectional forwarding graph construction, and 3) saturation attack detection. The description of these steps is shown below.

- Step 1: Graph information preprocessing. TITAN first gives the definitions of flow forwarding rules and topology information. Then it designs flow statistical features. The flow forwarding rules, topology information and flow statistical features are delivered to the next step to generate graph.
- Step 2: Bidirectional forwarding graph construction. TITAN proposes BiFor-Graph, a bidirectional forwarding graph construction algorithm. BiFor-Graph first constructs the basic graph structure of the bidirectional forwarding graph for each network flow. Subsequently, it generates the node information based on the statistical characteristics of the network flow at different switches.
- Step 3: Saturation attack detection. TITAN constructs a GCN model to detect the saturation attack. The bidirectional forwarding graph generated in step 2 is fed into that saturation attack detection model to perform graph detection and determine whether the network flow is a saturation attack flow.

### 4.2 Graph information preprocessing

TITAN utilizes graphs to represent network flows based on the information of network flows and network topology. The SDN controller has a global view over the entire network [28]. Therefore, as shown in Fig 2, TITAN takes advantage of the SDN controller to obtain the network topology, flow forward rules, and statistical characteristics of network flow to generate bidirectional forwarding graphs for traffic flows. The variables used in the procedure of generating the BiFor-Graph are shown in Table 2.

**Table 2:** Definition of variables.

Variable	Value
L	Topology Information Set
*ℓ*	Physical Link
*φ*_*s*_	Source IPv4 Address
*φ*_*d*_	Destination IPv4 Address
*φ*_*δ*_	Switch Dpid
*ψ*	Physical Port
Ω	Forward Rules Set
*χ*	Forward Rules of a Flow
*γ*	Forward Rules of a Flow in a Switch

To generate a bidirectional graph, TITAN gives two definitions, including 1) topology information L and 2) flow forward rules Ω.

**Definition 1**. Topology information L. This work designs a tuple $L=\{\ell_{1},\ldots ,\ell_{k}\}$ to represent the topology information.

$$\begin{array}{c} \ell_{i}=(\varphi_{p},\psi_{p})↔(\varphi_{q},\psi_{q}) \end{array}$$ (1)

Here, *ℓ*_*i*_ is the link between a host and a switch or the link between two switches. *φ*_*p*_ and *φ*_*q*_ are IP addresses of hosts or IDs of switches.

**Definition 2**. Flow forward rules Ω. This work utilizes a tuple Ω = {*χ*_1_, …, *χ*_*m*_} to represent the flow forward rules.

$$\begin{array}{c} \chi_{i}=\{\varphi_{s},\varphi_{d},\gamma_{1},\ldots ,\gamma_{n}\},i=1,2,\ldots ,m \end{array}$$ (2)

$$\begin{array}{c} \gamma_{j}=(\varphi_{\delta }^{j},\psi_{\text{out}}) \end{array}$$ (3)

*φ*_*s*_ is the source IP address. *φ*_*d*_ is the destination host IP address. $\varphi_{\delta }^{j}$ is the switch and *ψ*_out_ is the outgoing port. *γ*_*j*_ is the forwarding rule. *γ*_*j*_ means that in switch $\varphi_{\delta }^{j}$, the packets with source IP *φ*_*s*_ and destination IP *φ*_*d*_ should be forwarded via port *ψ*_out_. Each traffic flow has one or more forwarding rules, therefore *χ*_*i*_ = {*φ*_*s*_, *φ*_*d*_, *γ*_1_, …, *γ*_*n*_} means that the flow from *φ*_*s*_ to *φ*_*d*_ is forwarded by *n* switches.

Based on our previous research [18, 29, 30], TITAN extracts five flow-based statistical features to detect saturation attack flow. Usually, the saturation attack against an SDN switch tries to deplete the flow entry storage of the switch. Hence, it can prevent the switch from installing new flow entries and translating new packets. Unlike traditional momentary and burst DDoS attacks, the attacker needs to send attack flows to the target switch periodically to add new flow entries and lengthen the survival time of these flow entries. This means that the saturation attack flow against the switch has two main properties, the periodicity and the longer survival time of the flow entries. More specially, the saturation attack tries to make the abnormal flow entries always exist in the switch, while the normal flow table entries are usually deleted due to the timeout. So the survival time of the saturation attack flow is longer than that of the normal flow entries. With the above analysis, TITAN extracts 5-dimensional statistic features *f* = {*α*, *V*_*α*_, *β*, *V*_*β*_, *τ*} as the main statistical features to generate the bidirectional forwarding graph.

- *α*. The total packet number of a traffic flow. In SDN, a new arrival packet will make the controller install a new flow entry on the switch. And the goal of saturation attack is to occupy the flow table space. Besides, the attacker often send less malicious packets in order to make the saturation attack more stealthy [31]. Hence, there is a difference in the total packet number between the normal flow and attack flow.
- *V*_*α*_. The packet rate of a network flow during a sampling interval. *T* is the current moment and Δ*t* is the sampling period. The saturation attack makes the malicious flow entries alive by sending attack flows periodically. However, the communication of normal terminals is irregular. Therefore, there is a difference in the packet rate between the normal flow and attack flow.
  $$\begin{array}{c} V_{\alpha }=\frac{\alpha^{T}-\alpha^{T-\Delta t}}{\Delta t} \end{array}$$ (4)
- *β*. The total byte number of a traffic flow. Similarly, when launching a saturation attack, each attack flow carries a small length of data for concealing the attack traffic [31]. However, the byte number of normal flow is irregular. Hence, the total byte number of a flow can be utilized to identify the normal flow and attack flow.
- *V*_*β*_. The byte rate of a network flow during a sampling interval. According to the analysis of the packet rate of a network flow, the byte rate of attack flow and normal flow show a similar difference with the total byte number of a traffic flow. Therefore, the byte rate of a network flow during a sampling interval can also be employed to detect attack flows.
  $$\begin{array}{c} V_{\beta }=\frac{\beta^{T}-\beta^{T-\Delta t}}{\Delta t} \end{array}$$ (5)
- *τ*. The survival time of a flow. The goal of a saturation attack against SDN switches is to occupy the flow table storage capacity. The adversary needs to send malicious packets before the malicious flow entry idle timeout arrives to lengthen the survival time of the malicious flow entry. Hence, the survival time of attack flow entries is longer than normal flow entries. In other words, the survival time of a flow is a representation of the saturation attack flows.

**Algorithm 1** BiFor-Graph.

**Input**: L,Ω,F

**Output**: *Graph* = {*G*_1_, …, *G*_*m*_}

 **for** *χ*_*i*_ ∈ Ω **do**

  **generate empty graph** *G*_*i*_ = Γ(*V*, *E*, *F*)

  *V* ← *v*_0_(*F*(*φ*_*s*_))

$\theta \leftarrow \nabla (\varphi_{s},\psi_{s},L)$

  *V* ← *v*_1_(*F*(*θ*, *φ*_*s*_ → *φ*_*d*_))

  *E* ← < *v*_0_, *v*_1_ >

  **for** *γ*_*j*_ ∈ *χ* **do**

   **if** $\gamma_{j}.\varphi_{\delta }^{j}==\theta$ **then**

$\theta \leftarrow \nabla (\gamma_{j}.\phi_{\delta }^{j},\gamma_{j}.\psi ,L)$

    *V* ← *v*_*j*+1_(*F*(*θ*, *φ*_*s*_ → *φ*_*d*_))

    *E* ← < *v*_*j*_, *v*_*j*+1_ >

   **end if**

   **until the** *v*_*n*_ **of** *φ*_*d*_ **is inserted to** *V*

   **inserting the reverse path of the flow into** *G*_*i*_

  **end for**

  *Graph* ← *G*_*i*_

 **end for**

 **return** *Graph*

Then, TITAN utilizes *f* to represent a flow, and *F* = {*f*_1_, …, *f*_*k*_} is the set of all flow.

After preprocessing the topology information L, flow forward rules Ω, and flow statistical features *f*, TITAN will deliver the above data to the second step to construct the BiFor-Graphs.

### 4.3 Bidirectional forwarding graph construction

TITAN designs BiFor-Graph, a bidirectional forwarding graph generation algorithm, with the definitions of topology information L, flow forwarding rules Ω and flow statistical characteristics *F* = {*f*_1_, …, *f*_*k*_} in Section 4.2. BiFor-Graph translates each flow of the network to bidirectional forwarding graphs. As shown in Algorithm 1, at first, for each *χ*_*i*_ in Ω, it generates an empty graph *G*_*i*_ = (*V*, *E*, *F*) for each flow by applying the function Γ. Here, *V* is the node set and *E* is the edge set.

$$\begin{array}{c} G_{i}=\Gamma \left(V,E,F\right),V=\emptyset ,E=\emptyset \end{array}$$ (6)

After obtaining the empty graph *G*_*i*_, BiFor-Graph gets the nodes and edges information of the graph. It first insert *v*_0_ corresponding to the source host *φ*_*s*_ and its node information *F*(*φ*_*s*_) into *V*. BiFor-Graph searches the next hop using Eq 7. This function can extract the next-hop based on the topology information L and flow forwarding rules Ω. After obtaining the next hop of *φ*_*s*_, BiFor-Graph starts to generate other nodes and links in sequence based on L and Ω. Specially, the next hop *v*_1_ of *v*_0_ is generated using Eq 8. Then *v*_1_ is inserted into *V* and the related link < *v*_0_, *v*_1_ > is inserted into *E*. For each node, The search process will continue until the destination host *φ*_*d*_ is found.

$$\begin{array}{c} \theta =\nabla \left(\varphi_{s},\psi_{s},L\right),\left(\varphi_{s},\psi_{s}\right)↔\left(\varphi_{\delta }^{1},\psi_{1}\right)\in L \end{array}$$ (7)

$$\begin{array}{c} \theta =\{\begin{array}{c} \varphi_{d},(\varphi_{\delta }^{j},\psi_{j}^{\text{out}})↔(\varphi_{d},\psi_{d})\in L\\ \varphi_{\delta }^{j+1},(\varphi_{\delta }^{j},\psi_{j}^{\text{out}})↔(\varphi_{\delta }^{j+1},\psi_{j+1})\in L \end{array} \end{array}$$ (8)

Up to now, the nodes and edges from *φ*_*s*_ to *φ*_*d*_ have been completely loaded into the *G*_*i*_. In the next step, the path from *φ*_*d*_ to *φ*_*s*_ is added to the *G*_*i*_. Ultimately, the BiFor-Graph writes node information to the graph based on the flow statistical features *F* to obtain a bidirectional forwarding graph of the current traffic flow.

**Fig 3:**
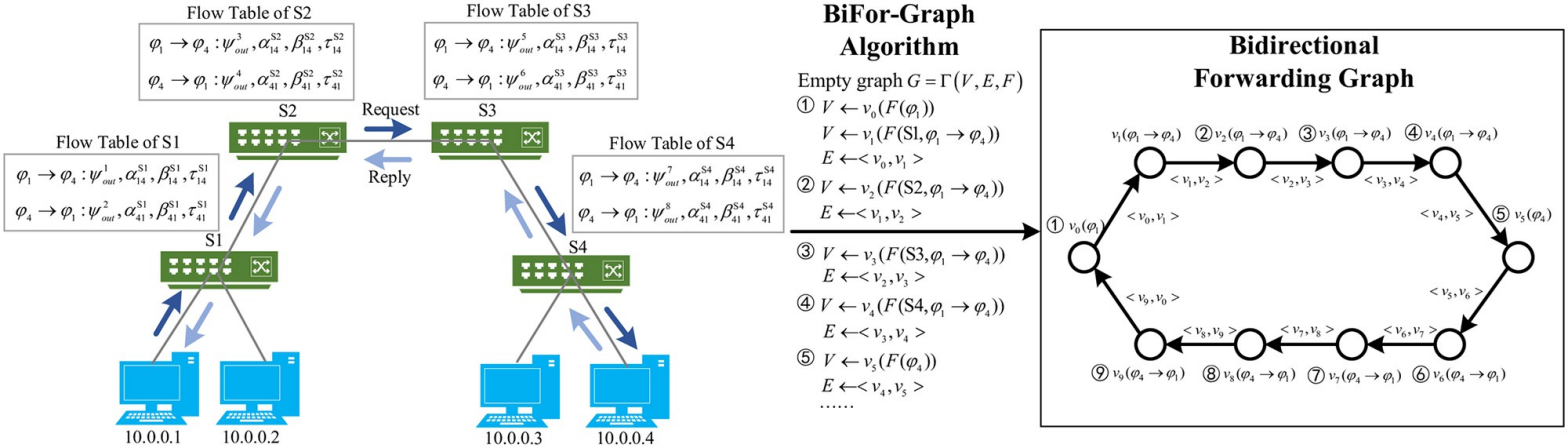
Construction of bidirectional forwarding graph.

For a saturation attack flow, suppose that its statistical features on each switch have been calculated as *F*. We give an example for describing the constructing process of Bifor-graph in Fig 3. Assuming that *φ*_1_(10.0.0.1) is sending packets to *φ*_4_(10.0.0.4). The relevant flow entries are installed on S1, S2, S3, and S4. TITAN will first preprocess the topology information, flow forward rules and statistical features. Subsequently, TITAN constructs bidirectional forwarding graph using the BiFor-Graph algorithm based on the pre-processed data. Specially, BiFor-Graph generates an empty graph *G* = (*V*, *E*, *F*). Then it inserts the source node *v*_0_ of source host *φ*_1_ and its node information *F*(*φ*_1_) into *V*. Subsequently, it searches the next-hop of *φ*_1_, < *v*_1_ >. Then the information about the switch S1 is calculated and assigned as the value of < *v*_1_ >. The link < *v*_0_, *v*_1_ > between the two nodes is generated and added into *E*. BiFor-Graph continues to search for nodes that the flow passes through until the destination host *φ*_4_ is searched. Then it start to find and add the reverse path of the flow to *G*. Finally, we obtain a bidirectional forwarding graph of the flow between *φ*_1_ and *φ*_4_.

Then, TITAN will feed the BiFor-Graphs to the detection model for identifying saturation attack flows.

### 4.4 Saturation attack detection based on BiFor-Graph

**Fig 4:**
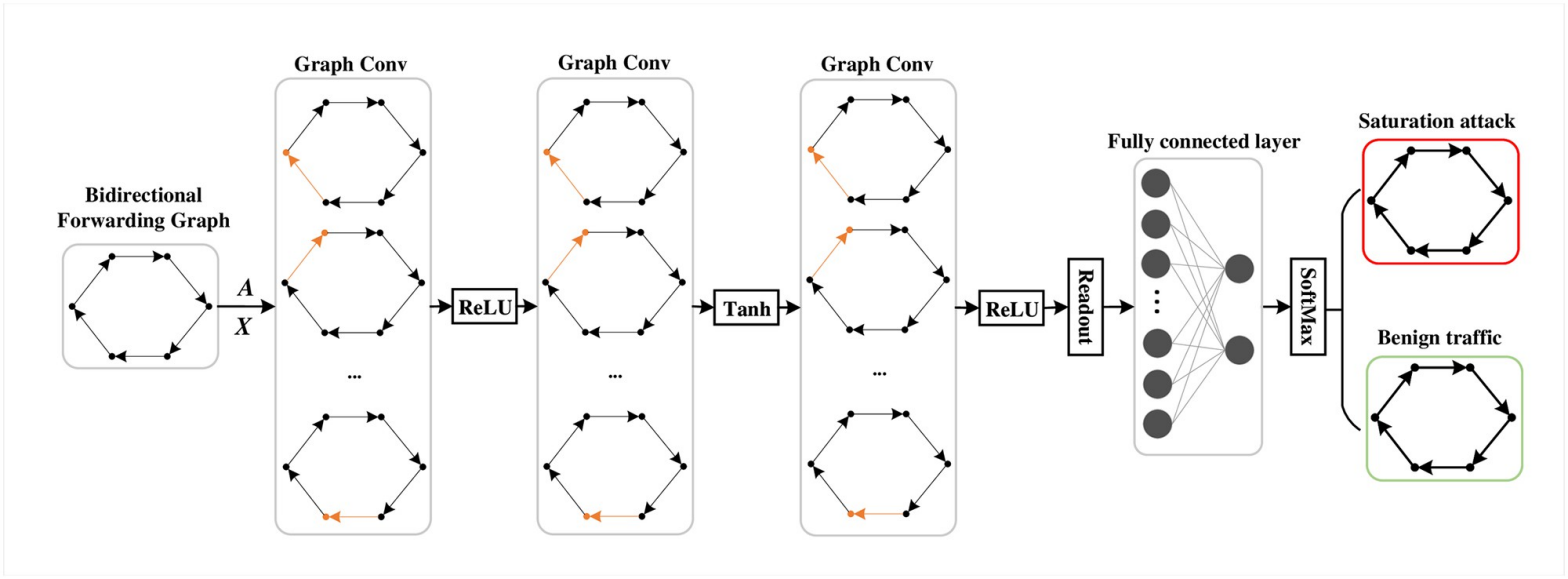
Detection model of saturation attacks based on GCN.

For a traffic flow, after generating a bidirectional forwarding graph *G* = (*V*, *E*, *F*), TITAN utilizes GCN to determine whether the bidirectional forwarding graph is saturation attack. TITAN aggregates the neighbor node information to the central node based on the graph structure to improve the representability of the graph. As shown in Fig 4, the saturation attack detection model consists of three graph convolutional layers, one fully connected layer, and one classification module. At first TITAN extracts the adjacency matrix *A*, degree matrix *D* and feature matrix *X* from *G*. Then the detection model performs a convolution operation on a node in *G*. It first extracts the feature of the central node’s neighbors as convolution parameters. Then it merges the node’s own feature into the convolution operation. Finally, the saturation attack detection model represents a node using the combined features of itself and its neighbors. The graph convolution layer is described as Eq 9.

$$\begin{array}{c} H^{l+1}=\sigma \left({\tilde{A}}^{-\frac{1}{2}}\tilde{A}{\tilde{D}}^{-\frac{1}{2}}H^{l}W^{l}\right) \end{array}$$ (9)

In Eq 9, *σ* is the active function. $\tilde{A}=A+\lambda \times I_{N}$(λ = 1) denotes the adjacency matrix *A* plus the identify matrix after adding the self-loop for each node. $\tilde{D}$ indicates the degree matrix after adding the self-loop. *W*^*l*^ means the weight matrix of the *l*-th convolution layer, while *H*^*l*^ is the feature matrix calculated after the operation of the *l*-th graph convolution layer. In particular, *H*^0^ = *X*.

With three graph convolution layers, the model learns a more comprehensive representation for each node in *G*. It is followed by aggregating the features of each node as the graph feature using the *Readout* function shown in Eq 10. *H*^*n*^ = {${\vec{h}}_{1},{\vec{h}}_{2},...,{\vec{h}}_{N}$} is the node feature matrix of *G* that will be processed by the *n*-th graph conv layers. More specifically, *N* is the node number and ${\vec{h}}_{i}$ is the feature of *i*-th node. In Eq 10, *Feat*_*G*_ is obtained by calculating the mean value of all node features in input graph *G*.

$$\begin{array}{c} Feat_{G}=\frac{1}{N}\sum_{i=0}^{N}{\vec{h}}_{i} \end{array}$$ (10)

After obtaining the graph feature *Feat*_*G*_ by *Readout*, the *Feat*_*G*_ is input into the fully connected layer for calculating the probability of whether the bidirectional forwarding graph is saturation attack. And (*y*_0_,*y*_1_) is the output of the fully connected layer. Then we minimize the loss function cross-entropy between *y*_*j*_ and the original label *y* to optimize this model:

$$\begin{array}{c} Loss=-\sum_{j=0}^{1}y\cdot {\text{log}}_{2}\;y_{j} \end{array}$$ (11)

During the saturation detection, the final output probability *y*_*j*_ is mapped to the (0, 1) using *Softmax*, and the value can be considered as the probability that the instance is a saturation attack. The *Softmax* is shown in Eq 12.

$$\begin{array}{c} f\left(y_{j}\right)=\frac{e^{y_{j}}}{e^{y_{0}}+e^{y_{1}}} \end{array}$$ (12)

Finally, TITAN uses *argmax* function to identify whether the bidirectional forwarding graph belongs to attack instance. The *argmax* will output the category with the maximum in *f*(*y*_0_) and *f*(*y*_1_). At last, *predict*_*G*_, the detection result of bidirectional forwarding graph *G* is calculated by Eq 13.

$$\begin{array}{c} \begin{array}{cc} predict_{G} & =argmax(f\left(y_{j}\right))\\ & =\{y_{j}|\forall y_{k}:f\left(y_{k}\right)\leq f\left(y_{j}\right)\} \end{array} \end{array}$$ (13)

## 5 Experiments and evaluation

This section describes the evaluations of TITAN with three subsections, namely, Experiment Setup (subsection 5.1), Changes of Features (subsection 5.2) and Saturation Attack Detection Performance Comparison (subsection 5.3).

### 5.1 Experiment setup

**Fig 5:**
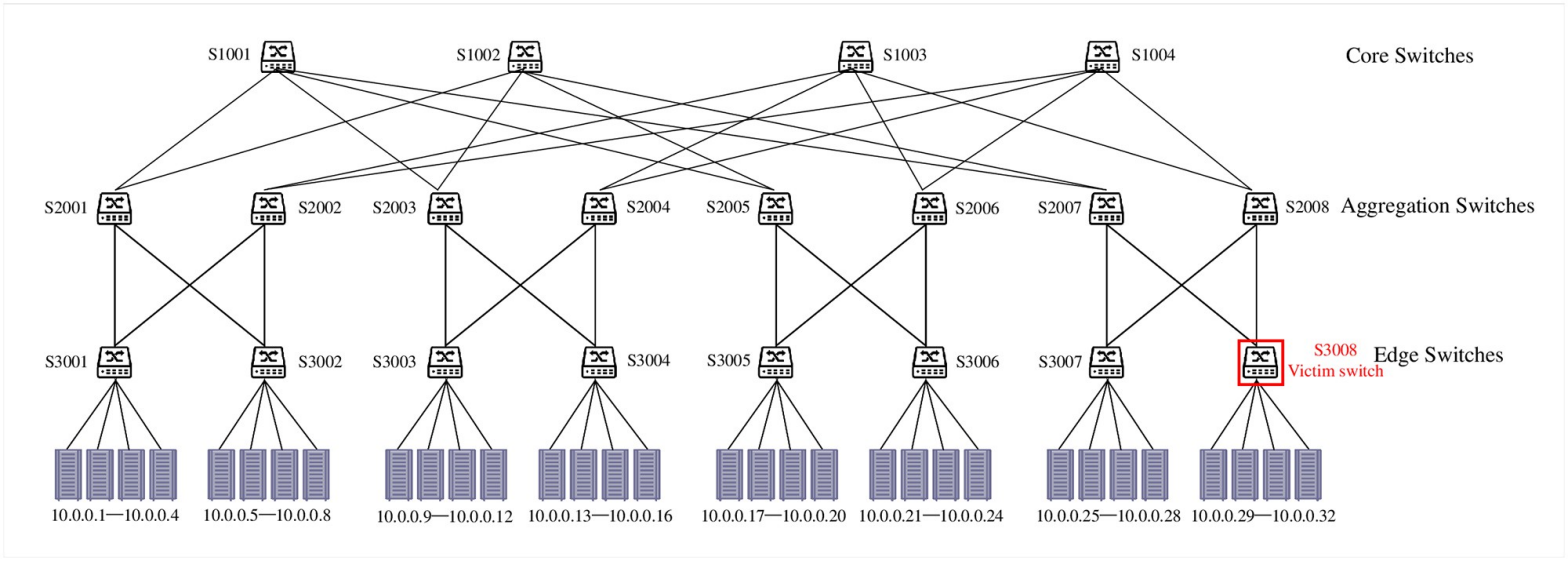
Experiment topology.

We select the Ryu 1.4 as the SDN controller. For hosts and switches, this evaluation employes Mininet 2.3.0 for simulation according to the analysis about Mininet [32]. Simultaneously, it utilizes the OpenFlow 1.3 protocol as the southbound interface communication protocol. To verify the effectiveness of TITAN, we construct the Fat-tree topology that contains twenty switches and thirty-two hosts, as shown in Fig 5. More specially, the Fat-tree topology consists of four core switches(S1001-S1004), eight aggregation switches(S2001-S2008) and eight edge switches(S3001-S3008). Besides, each edge switch is connected to four hosts.

For verifying the effectiveness of TITAN, this evaluation simulates a saturation attack targeting at SDN switches. In the experiment, eight hosts are randomly selected to send TCP, UDP, and ICMP data packets to each other to simulate background traffic as our previous works [18, 29, 30]. In this evaluation, we try to attack the switch S3008. Hence, we randomly selects four hosts as the saturation attackers and make these hosts send saturation attack traffic to hosts 10.0.0.29, 10.0.0.30, 10.0.0.31, and 10.0.0.32. The attack traffic causes S3008 to install the malicious flow entries. The attacker will also periodically inject the attack traffic into the network to prolong the survival time of the malicious flow entries. Eventually, the flow table storage of S3008 overflows and the normal communication of the network is affected. To verify the effectiveness of TITAN, this evaluation sets 9 attack traffic proportions, from 10% to 90%. The attack traffic proportions is the percentage of attack traffic accounts for total network traffic.

The GCNID [11], GCN-TC [12], Logistic Regression (LR), Naive Bayes (NB), and Support Vector Machines (SVM) algorithms are selected as the comparison algorithms in this evaluation. GCNID generates one KNN graph for each flow. Then the KNN graph is sent to the GCN model for detecting saturation attack. GCN-TC produces a traffic trace graph. Its nodes represent the traffic flows and its edges denotes the nodes have common IP address. Saturation attack detection is performed by analyzing the nodes in that graph. The LR, NB, and SVM algorithms utilize the flow statistical features for performing saturation attack detection.

### 5.2 Changes of features

Subsequently, we will analyze the trend of the statistical features selected by TITAN with respect to attack and normal traffic.

#### 5.2.1 Average packets rate and bytes rate

**Fig 6:**
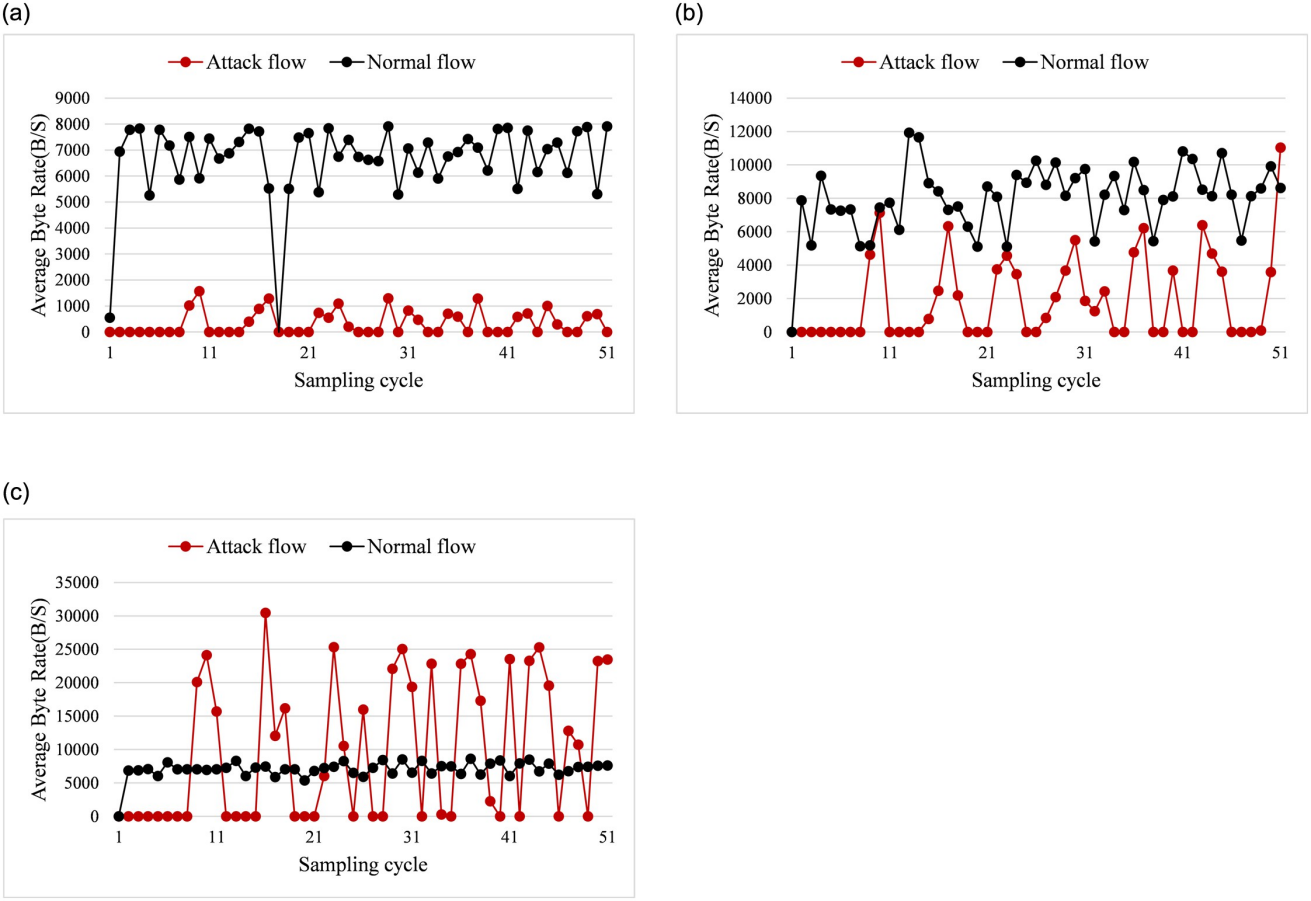
Average byte rate. (a) attack traffic proportion = 10%. (b) attack traffic proportion = 40%. (c) attack traffic proportion = 80%.

**Fig 7:**
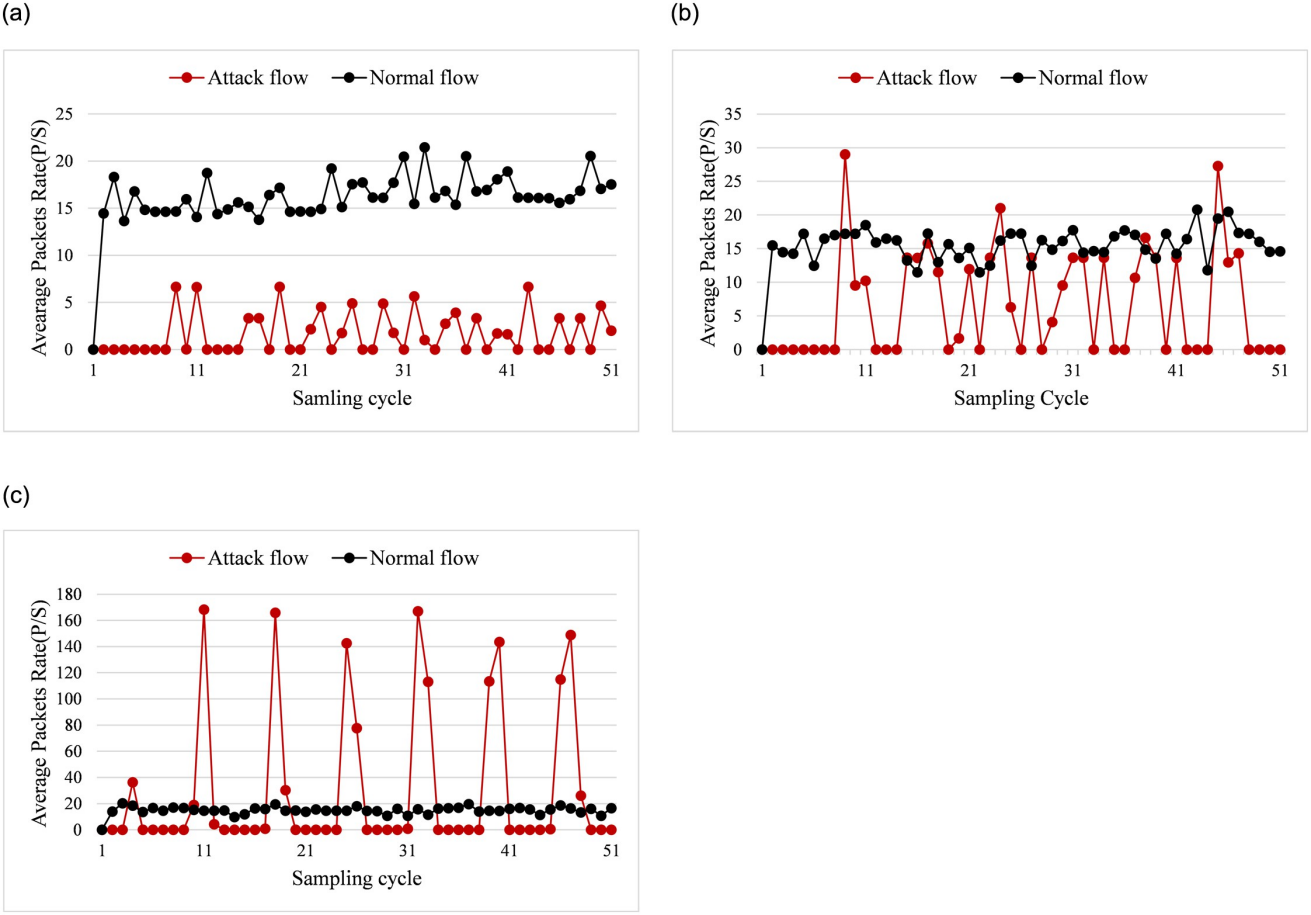
Average packets rate. (a) attack traffic proportion = 20%. (b) attack traffic proportion = 50%. (c) attack traffic proportion = 90%.

Figs 6 and 7 show the changes of average bytes rate and packets rate. Fig 6 illustrates the changes of average byte rate when the saturation attack traffic proportion are 10%, 40%, and 80%. Fig 7 presents the variation of average packet rate as the attack traffic are about 20%, 50%, and 90%.

From Fig 6, we can see that when the saturation attack traffic proportion are 10%, and 80%, the average bytes rate differs significantly from that of normal traffic. However, when the attack traffic proportion is 40%, the byte rate of attack flow is similar with the normal flow. From Fig 6a, the maximum byte rate of the attack flow is close to 2 thousands bytes per second when the total flow ratio is 10%. The interval between each attack is 1 to 4 cycles, which shows a regular change. In Fig 6b, when the attack traffic proportion is increased to 40%, the byte rate of attack flow is close to that of the normal traffic. The reason for this phenomenon is that the saturation attack traffic improves to 40%, the normal traffic accounts for 60% of the total network traffic. Hence the byte rate of attack and normal traffic is similar. When the attack ratio is raised to 80%, the byte rate of the attack traffic can reach up to about 30 thousands bytes per second, which is quite bigger than normal traffic, as seen in Fig 6c. Since the saturation attack is launched through multiple threads in the simulated experiment, the period of the attack may not be consistent for each thread, and results in the period of the attack being less regular in Fig 6c.

Fig 7 illustrates the variation of average packets rate when the attack traffic proportion are 20%, 50%, and 90%. When the attack traffic proportion is 20%, the average packet rate of normal traffic is higher than attack traffic from Fig 7a. If the attack traffic proportion increases to about 50%, the packet rate of the attack traffic is close to the normal traffic for most cycles. As the attack traffic proportion raises to 90%, the attack flow has higher average packet rate than normal traffic. Meanwhile, the saturation attack also shows more clear periodicity.

In conclusion, from Figs 6 and 7, we can conclude that the average byte rate and packet rate of normal traffic changes randomly and irregularly. On the other hand, the saturation attack traffic is always injected into the network periodically. When the attack traffic proportion is low or high, it is easy to detect the saturation attack flow. However, when the proportion of attack traffic is 40% or 50%, the difference of average byte rate and packet rate of the attack traffic and normal traffic is not significant. In this case, identifying attack flows from the normal flow becomes more difficult.

#### 5.2.2 Lifetime of flow entry

**Fig 8:**
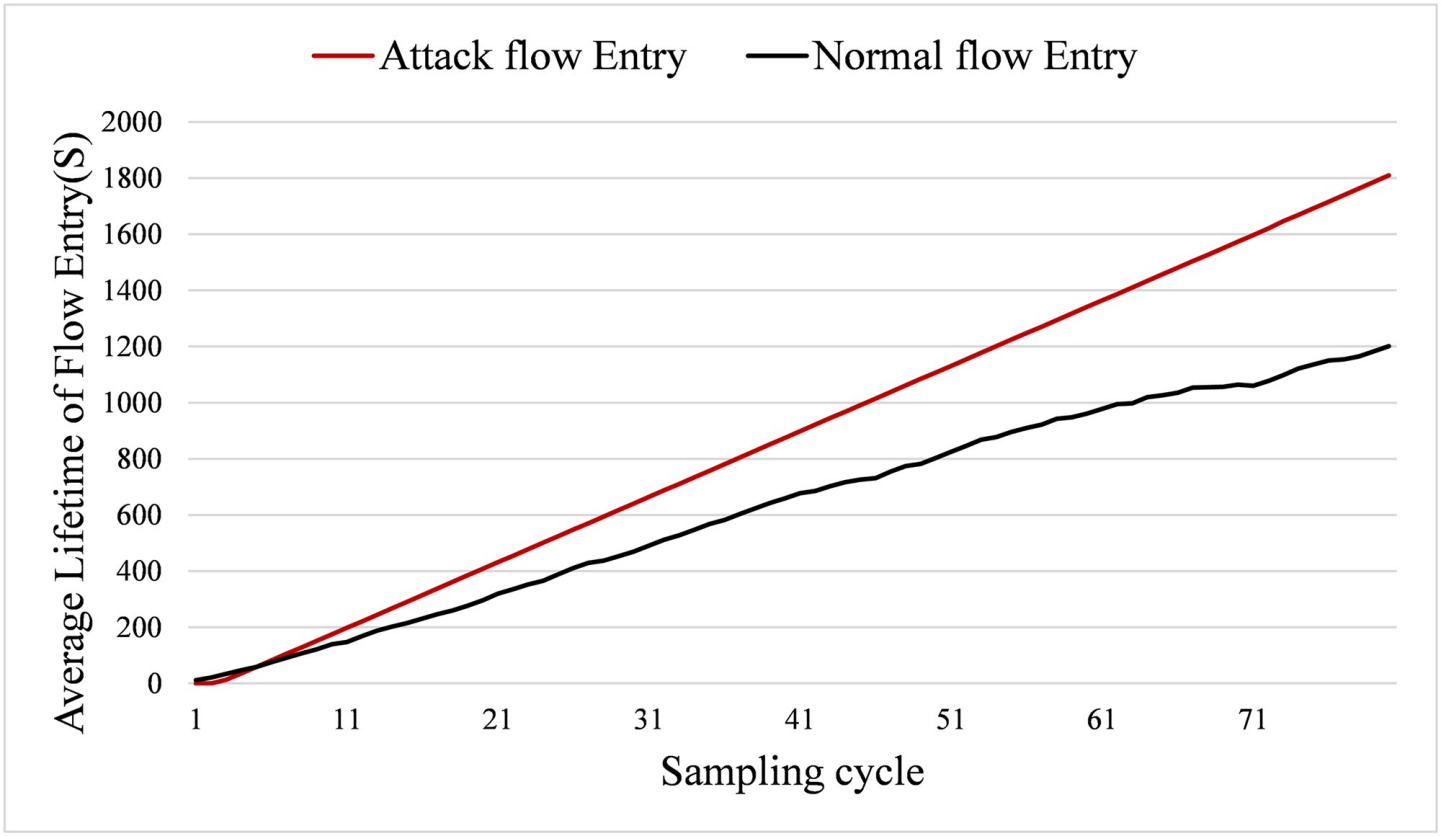
Average lifetime of flow entry.

Fig 8 shows the comparison of the average survival time of normal flow entries and attack flow entries. From Fig 8, it is shown that the averagy lifetime of the attack flow entries is slightly lower than normal in the earlier cycles because the start time of the attack traffic is later than normal traffic. After a few cycles, the attack traffic is injected into the network and the OpenFlow switch installs the relevant flow entries. With the continued attack traffic, the growth rate of the attack flow entry’s lifetime starts to increase with a higher rate than normal flow entries.

The reason of this phenomenon is that the normal traffic in this evaluation is randomly launched, and the OpenFlow switch installs flow entry for newly traffic using idle timeout. In this case, the normal flow entries may be deleted due to the overtime of idle timeout. However, for the saturation attack, it will try its best to make the attack flow entries survival long time in the switch. Hence, it will periodically send attack traffic into the network to lengthen the lifetime of the attack flow entries. Therefore, the average lifetime of attack flow entries keeps growing, with a higher speed than normal flow entries.

For instance, in the 70th cycle, the survival time of normal flow entries reaches 1000s and then a pause occurs. At this point, the average survival time of normal flow entries stops growing as some normal flow entries reach the idle timeout and these flow entries are deleted. Then the switch installs new flow entries for the relevant traffic flows. However, the survival time of the attack flow entries always increases linearly. At the 80th cycle, the average survival time of the attack flow entries grows to 1800s, while the survival time of the normal flow entries reaches only 1200s.

In conclusion, the survival time of attack flow entries is higher than normal flow entries from Fig 8. Hence, utilizing the survival time of a flow entry to detect saturation attack flow may improve the detection performance.

### 5.3 Saturation attack detection performance comparison

**Fig 9:**
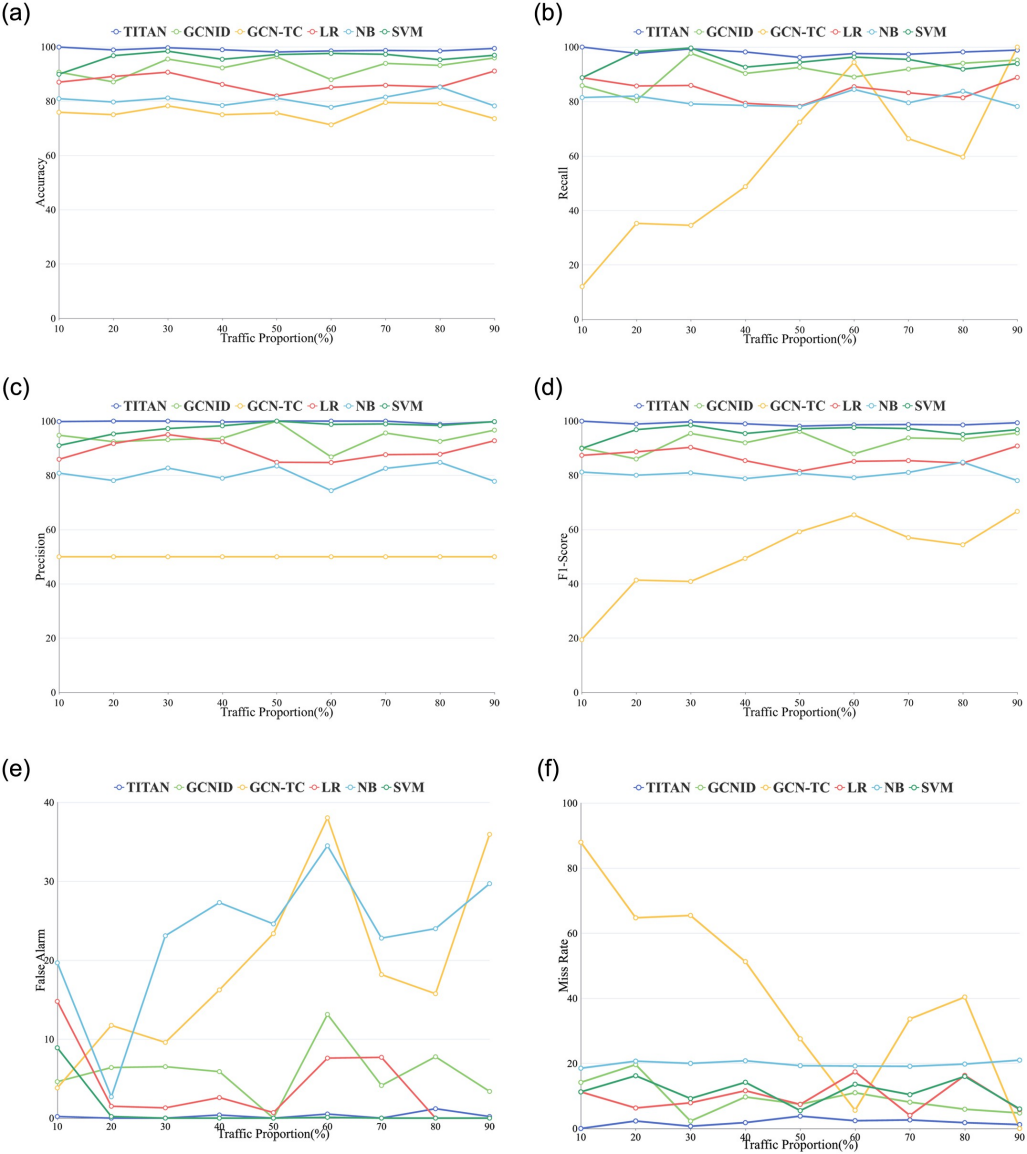
Detection performance comparison under nine traffic proportions. (a) Accuracy. (b) Recall. (c) Precision. (d) F1-Score. (e) False Alarm. (f) Miss Rate.

**Fig 10:**
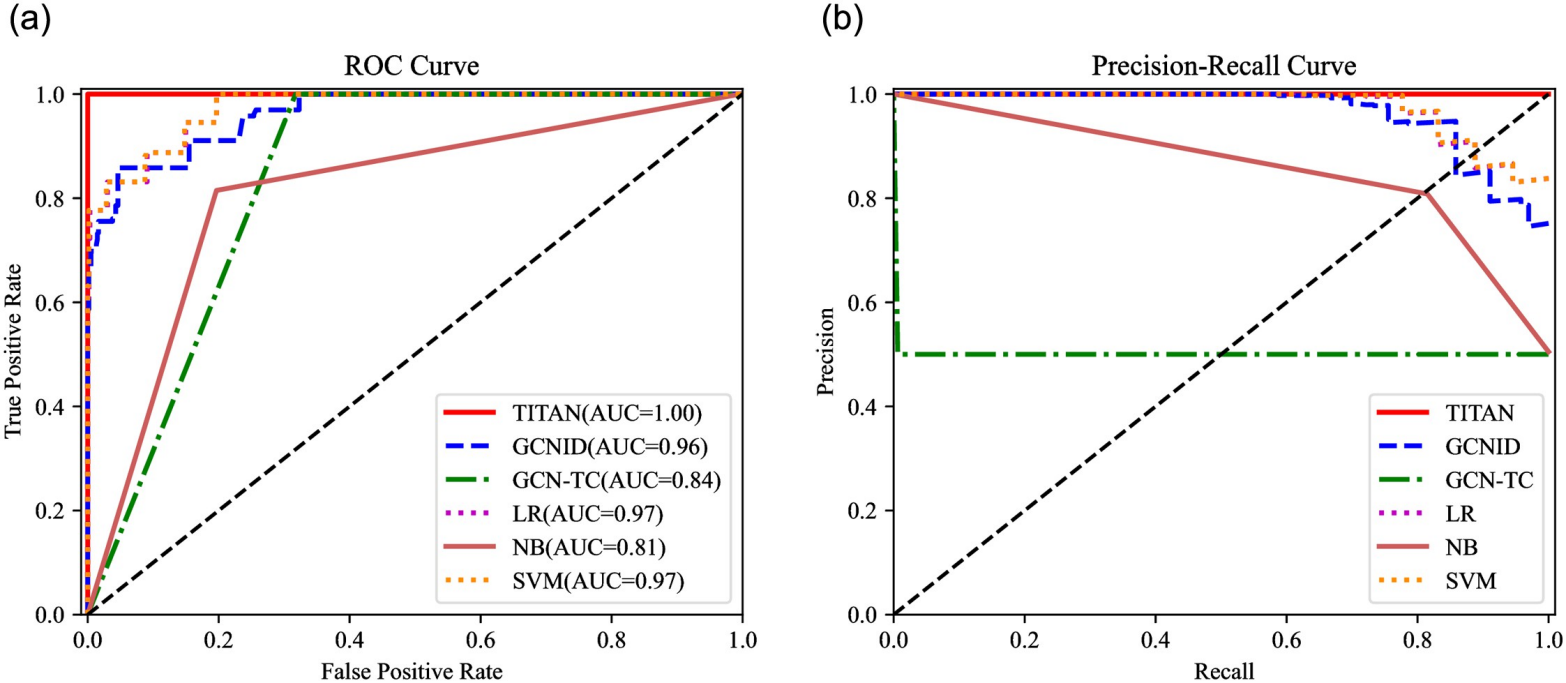
ROC curve and P-R curve (attack traffic proportion = 10%). (a) ROC curve. (b) P-R curve.

**Fig 11:**
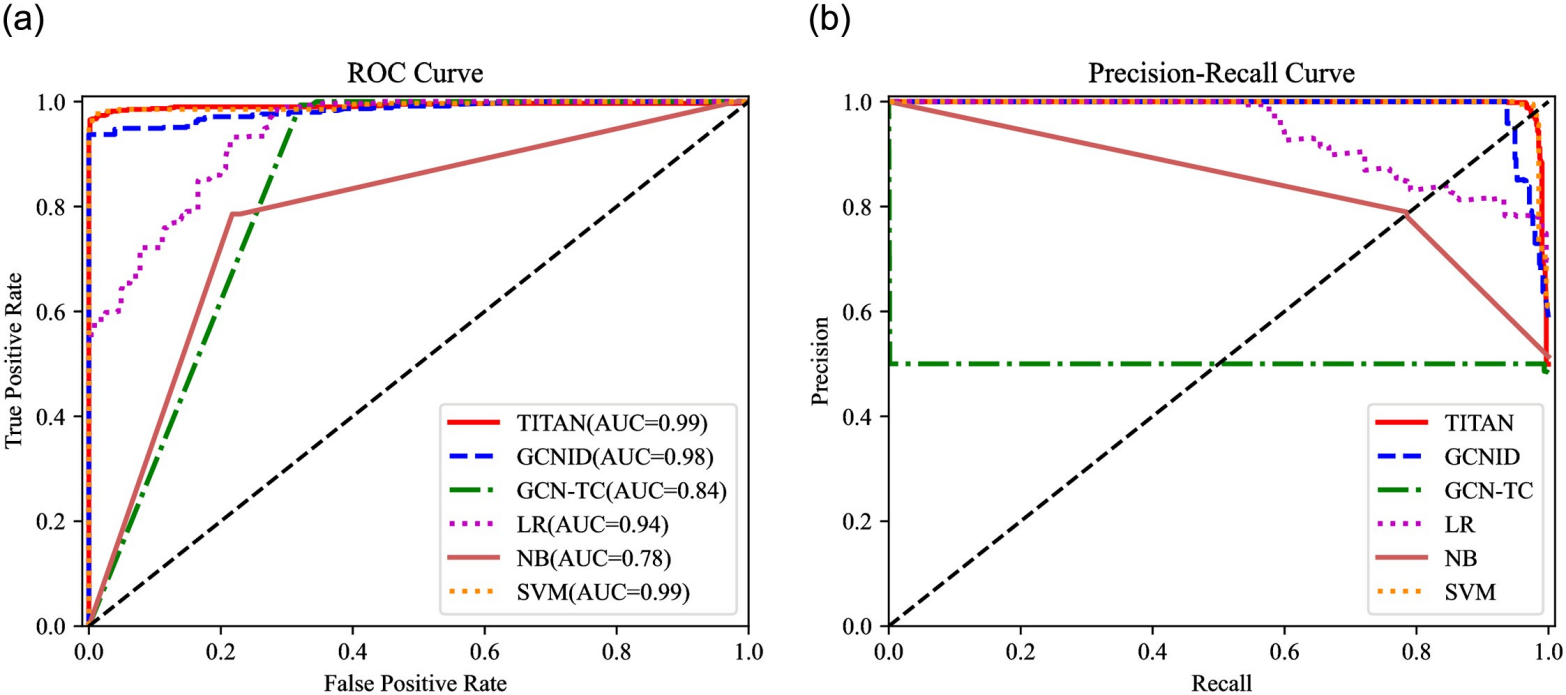
ROC curve and P-R curve (attack traffic proportion = 50%). (a) ROC curve. (b) P-R curve.

**Fig 12:**
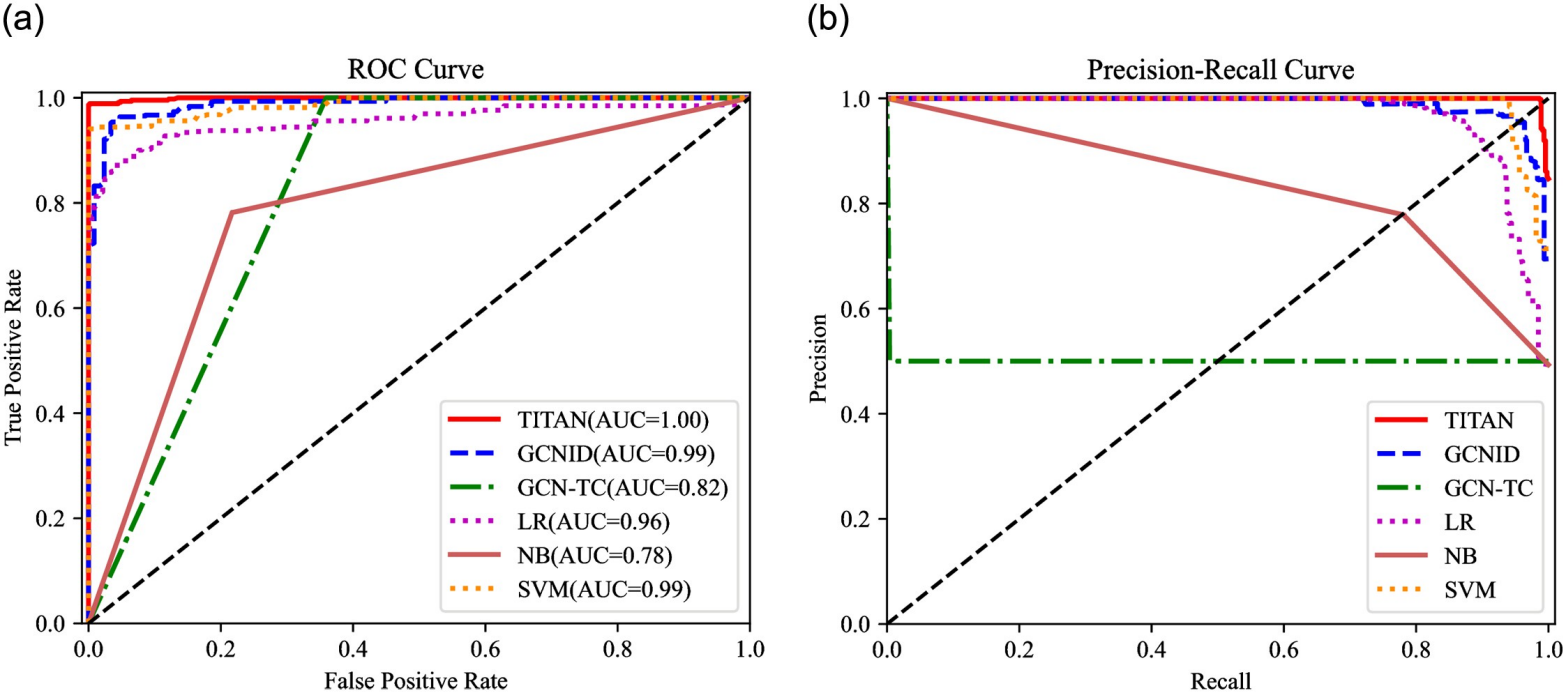
ROC curve and P-R curve (attack traffic proportion = 90%). (a) ROC curve. (b) P-R curve.

In this evaluation, the saturation attack detection performance of TITAN and the compared algorithms are evaluated. Eight metrics, including the Accuracy (Acc), Recall (Rec), Precision (Pre), F1-score (F1), Fasle Alarm (FA), Miss Rate (MR), ROC (Receiver Operating Characteristic) curves, and P-R (Precision-Recall) curves are utilized to evaluate TITAN, GCNID, GCN-TC, LR, NB, and SVM, as shown in Table 3, Figs 9–12.

**Table 3:** Detection performance comparison.

Attack traffic(%)	Evaluation metric	TITAN	GCNID	GCN-TC	LR	NB	SVM
10	Acc	**99.91**	90.66	75.90	87.00	80.91	89.91
	Rec	**100.0**	85.83	12.03	88.76	81.48	88.76
	Pre	**99.83**	94.78	50.00	85.92	80.81	91.01
	F1	**99.91**	90.08	19.39	87.31	81.15	89.87
	FA	**0.20**	4.62	3.82	14.79	19.67	8.91
	MR	**0.00**	14.17	87.97	11.24	18.52	11.24
20	Acc	**98.86**	87.08	75.00	89.08	79.66	96.75
	Rec	**97.71**	80.37	35.26	85.71	82.01	98.31
	Pre	**100.0**	92.41	50.00	91.72	78.08	95.27
	F1	**98.84**	85.97	41.35	88.61	80.00	96.77
	FA	**0.00**	6.41	11.75	1.50	2.70	0.20
	MR	**2.30**	19.63	64.74	6.30	20.70	16.20
30	Acc	**99.67**	95.50	78.26	90.66	81.16	98.41
	Rec	99.34	97.72	34.53	85.92	79.13	**99.66**
	Pre	**100.0**	93.15	50.00	95.05	82.69	97.25
	F1	**99.67**	95.38	49.35	90.26	80.87	98.44
	FA	**0.00**	6.52	9.59	1.30	23.10	**0.00**
	MR	**0.70**	2.28	65.47	7.90	20.00	9.20
40	Acc	**98.94**	92.25	75.00	86.16	78.41	95.41
	Rec	**98.21**	90.31	48.72	79.37	78.55	92.63
	Pre	**99.66**	93.65	50.00	92.38	78.94	98.26
	F1	**98.93**	91.94	49.35	85.38	78.74	95.36
	FA	0.40	5.89	16.24	2.60	27.30	**0.00**
	MR	**1.80**	9.69	51.28	11.60	20.80	14.20
50	Acc	**98.11**	96.33	75.61	81.91	81.08	97.16
	Rec	**96.21**	92.54	72.43	78.25	78.08	94.39
	Pre	**100.0**	**100.0**	50.00	84.82	83.45	**100.0**
	F1	**98.07**	96.12	59.16	81.41	80.68	97.11
	FA	**0.00**	**0.00**	23.37	0.70	24.60	**0.00**
	MR	**3.80**	7.46	27.56	7.40	19.30	5.50
60	Acc	**98.51**	87.91	71.28	85.08	77.75	97.58
	Rec	**97.63**	89.01	94.39	85.45	84.44	96.32
	Pre	**100.0**	86.79	50.00	84.74	74.37	98.79
	F1	**98.56**	87.88	65.37	85.09	79.09	97.54
	FA	0.52	13.14	38.04	7.60	34.50	**0.10**
	MR	**2.40**	10.99	5.60	17.40	19.20	13.60
70	Acc	**98.68**	93.91	79.49	85.83	81.50	97.25
	Rec	**97.37**	91.91	66.35	83.22	79.53	95.46
	Pre	**100.0**	95.62	50.00	87.63	82.57	98.95
	F1	**98.67**	93.73	57.02	85.37	81.02	97.18
	FA	**0.00**	4.13	18.18	7.70	22.80	**0.00**
	MR	**2.63**	8.09	33.65	4.03	19.10	10.40
80	Acc	**98.50**	93.16	79.09	85.25	85.16	95.25
	Rec	**98.20**	94.07	66.35	81.41	83.78	91.89
	Pre	**98.84**	92.55	50.00	87.79	85.81	98.37
	F1	**98.52**	93.31	54.38	84.48	84.78	95.02
	FA	1.19	7.77	15.76	**0.00**	24.00	**0.00**
	MR	**1.79**	5.92	40.39	16.30	19.80	16.00
90	Acc	**99.42**	95.91	73.56	91.08	78.25	96.91
	Rec	**98.88**	95.23	100.0	88.85	78.21	93.91
	Pre	99.77	96.66	50.00	92.76	77.81	**99.82**
	F1	**99.32**	95.54	66.67	90.76	78.01	96.77
	FA	0.20	3.38	35.94	**0.00**	29.70	**0.00**
	MR	1.20	4.77	**0.00**	5.80	21.00	6.00

The eight evaluation metrics are defined as follows.

$$\begin{array}{c} Acc=\frac{TP+TN}{TP+FP+FN+TN} \end{array}$$ (14)

$$\begin{array}{c} Rec=\frac{TP}{TP+FN} \end{array}$$ (15)

$$\begin{array}{c} Pre=\frac{TP}{TP+FP} \end{array}$$ (16)

$$\begin{array}{c} F1=2*\frac{Pre*Rec}{Pre+Rec} \end{array}$$ (17)

$$\begin{array}{c} FA=\frac{FP}{FP+TN} \end{array}$$ (18)

$$\begin{array}{c} MR=\frac{FN}{TP+FN} \end{array}$$ (19)

The horizontal axis of ROC curves is the FPR and the vertical is the TPR. AUC = (1+TPR-FPR)/2 is the area under the ROC curve, which ranges from 0 to 1. As AUC gets closer to 1, the more accurate the detection will be [33]. Additionally, Rec is the horizontal axis and Pre is the vertical axis in the P-R curve.

From Table 3, it can be seen that when the attack traffic proportion is about 50%, TITAN achieves the worst detection performance. However, most Acc, Rec, Pre, F1, FA, and MR values of TITAN are better than other methods.

For instance, when the attack traffic proportion is 20%, the accuracy of TITAN is 98.86%. However, the accuracy of GCNID, GCN-TC, LR, NB, and SVM are 87.08%, 75.00%, 89.08%, 79.66%, and 96.75%. In this case, TITAN can improve 13.5%, 31.8%, 11.0%, 24.1%, and 2.2% accuracy than GCNID, GCN-TC, LR, NB, and SVM. The FA and MR values of TITAN are 0.00% and 2.30%, respectively. The worst FA and MR are 11.75% and 64.74% for GCN-TC as the attack traffic proportion is 20%.

When the attack traffic proportion is low, TITAN also reaches better ROC and P-R curves than GCNID, GCN-TC, LR, NB, and SVM. For example, Fig 10 shows the ROC curves and P-R curves of TITAN, GCNID, GCN-TC, LR, NB, and SVM when the attack traffic proportion is 10%. From Fig 10, we can see that TITAN achieves the best ROC curve. More specially, as shown in Fig 10a, when FPR is 0, TPR of TITAN is about 100%, while TPR of GCNID, GCN-TC, LR, NB, and SVM are below to 80%.

For the medium attack traffic proportion, it is observed from the section 5.2 that with the attack traffic ratio rising to 40%, 50%, and 60%, both the packet rate and byte rate of attack and normal flow are closer to each other. Hence, we can observe from Table 3 that the detection accuracy of TITAN starts to decrease when the attack traffic proportion is 40%, and drops to a minimal 98.11% at attack ratio is 50%, and the MR increases to 3.8%. Meanwhile, for the three medium attack proportions, the Pre value of TITAN is 99.66%, 100.0%, and 100.0%, while the Rec value is 98.21%, 96.21%, and 97.63%, respectively. It demonstrates that distinguishing attack flows from normal flows is difficult in medium-ratio attacks.

We choose the ROC and P-R curves in Fig 11 when the attack traffic proportion is 50% to analyze the detection performance of TITAN. As shown in Fig 11b and Table 3, the Rec value of GCN-TC is 50% when the attack ratio is 50%. From Fig 11a, the AUC of both TITAN and SVM is 0.99, which is the highest among all algorithms. And the ROC and P-R curves of these two algorithms almost completely overlap. Therefore, from the two curves, we can conclude that TITAN and SVM have better performance in the medium proportions attacks. However, as shown in Table 3, TITAN also has better Acc, Rec, F1 FA and MR than SVM.

And with the attack traffic proportion increases to 70%, 80%, and 90%, the Acc of TITAN increases and is higher than the compared methods. When the proportion of attack traffic reaches 90%, the accuracy, recall, and precision of TITAN increases to 99.42%, 98.88%, and 99.77%, the false alarm and miss rate decreases to 0.20% and 1.20%. In the meantime, GCN-TC has better recall and SVM has larger precision value and a lower false alarm rate. However, the AUC of TITAN is bigger than all other methods. Meanwhile, the P-R curve of TITAN is higher than other algorithms from Fig 12b. Hence, in this case, TITAN has a better overall detection performance than other methods.

When the attack traffic proportion changes from 10% to 90%, TITAN’s false alarm rates are less than 0.5% most of the time, and the miss rates are lower than other methods. It shows that TITAN has fewer miss alarms and false alarms compared to other algorithms.

Here is the possible reason of that phenomenon. For GCNID, the generated *KNN* graph only contains information about the flow on a particular switch and lacks all the information about the flow in the network. It causes GCNID to fail to achieve better detection even the attack traffic proportion is low or high. In the meantime, the detection accuracy of GCN-TC are around 75%. Notably, the Rec of GCN-TC are very low and the Pre are all 50%. The possible reason for that is GCN-TC produces trace traffic graph by adding edges for nodes with common IP address. It makes some nodes that represent the reverse flow of attack flow only have the attack node as its neighbor node, while some nodes representing the attack flow also have only normal flow neighbor nodes. The GCN model will perform weighted average calculation on the features of the neighbor and the central nodes to obtain an average value as the central node’s feature. In this case, the GCN aggregates many attack nodes features in the trace traffic graph to a central normal nodes and also aggregates normal nodes features to attack node, resulting in a failure in detection.

For the LR algorithm, it is vulnerable to mistakenly detect normal traffic as attack. For the NB algorithm, it also cannot clearly distinguish attack traffic between normal traffic. In contrast, the SVM algorithm has a good detection result. However, when the proportion of attack traffic is increased to medium and high ratio, the recall of SVM is low, which means that SVM is difficult to identify the attack flow from the normal flows.

In conclusion, when the attack traffic proportion is low, medium, and high, TITAN can achieve higher accuracy, recall, precision, F1-score, false alarm and miss rate than other algorithms.

## 6 Conclusion

In this work, we propose a mechanism named TITAN for detecting saturation attack flows against SDN switches. Taking advantage of topology information, flow forward rules, and flow-based statistical features, TITAN designs BiFor-Graph, a bi-directional forwarding graph generation algorithm. The bi-directional forwarding graph contains the information of source, destination, forwarding path, and flow statistical features of a network flow. Furthermore, a GCN model is constructed for detecting saturation attack. We also made a detailed experiment for evaluating the detection performance of TITAN. The experiment results show that TITAN can effectively detect saturation attack against switches in SDN.

The experimental results show that TITAN outperforms other comparison algorithms because its generated graphs contain the bidirectional forwarding information for network flows, which has more comprehensive information than KNN graph. Additionally, compared with GCN-TC, TITAN can mitigate the negative effects on aggregating information caused by incorrectly connected edges.

This work proposes a BiFor-Graph-based mechanism to detect saturation attack towards SDN switches. However, the SDN controller is also the target of saturation attack. Consequently, it becomes challenging to simultaneously detect saturation attack flows targeting at the SDN controller and switches. Therefore, future research will focus on designing GNN-based methods to detect saturation attack flows targeted at SDN controller and switch. At the same time, TITAN is designed as a centralized saturation attack detection method. TITAN cannot detect saturation attack when it experiences runtime failures. Hence, in the future, we will also take our attention to distributed saturation attack detection methods.

In TITAN, we take the SDN controller to collect traffic statistics for detection. However, this method might lead to the controller-data bandwidth overhead. Consequently, there is a need for an attack detection data collection method that would not burden the network overhead. The network telemetry may be a suitable method to overcome that issue. Hence, the future work will focus on designing the network telemetry-based saturation attack detection method.
